# Epidemiology of hepatitis B and hepatitis C virus infections in pregnant women in Sana’a, Yemen

**DOI:** 10.1186/1471-2393-13-127

**Published:** 2013-06-07

**Authors:** Entisar A Murad, Suad M Babiker, Gasim I Gasim, Duria A Rayis, Ishag Adam

**Affiliations:** 1 Department of Obstetrics and Gynecology, AL Kuwait hospital, Sana’a, Yemen; 2 Faculty of Medicine, Alzaiem Alazhari University, Khartoum North, Sudan; 3 Faculty of Medicine, University of Khartoum, P O Box102, Khartoum, Sudan

## Abstract

**Background:**

Screening for Hepatitis B and C during pregnancy may help to decide on appropriate antiviral therapy and the institution of steps to minimize vertical transmission to the newborn infants.

**Methods:**

A cross-sectional study was conducted during November–December 2011 to investigate the seroprevalence and associated risk factors for markers of HBV (hepatitis B surface antigen; HBsAg) and anti-HCV antibody among pregnant women at the Al-Thawra hospital in Sana’a, Yemen. Structured questionnaires were used to obtain sociodemographic obstetrics and medical data and sera were tested for HBsAg and anti-HCV.

**Results:**

Of the 400 pregnant women enrolled in the study, HBsAg and anti-HCV were detected in 43 (10.8%; 95% CI: 8.0–14.0%) and 34 (8.5%, 95% CI: 6.0–11.5%) women, respectively. None of the women were co-infected with HBV and HCV. Multivariate analysis showed that circumcision was significantly associated with HBsAg seropositivity (OR = 3.3, 95% CI: 1.1–10.2; *p =* 0.03), low parity (primigravidae and secundigravidae) and education below secondary level were significantly associated with anti- HCV seropositivity (OR = 3.3, 95% CI: 1.1–10.2; *p =* 0.03). No other sociodemographic or clinical characteristics (age, residence, history of home delivery, miscarriage, dental manipulation, surgery, and blood transfusion) were significantly associated with HBsAg or anti-HCV seropositivity.

**Conclusion:**

The results of this study suggest that HBsAg and anti-HCV have high prevalence among pregnant women.

## Background

Developing countries have a high incidence of hepatitis B virus (HBV) and hepatitis C virus (HCV) infections [1,2], a fact that might partially be explained by non-adherence to known universal infection control procedures [3]. Women with hepatitis have an increased risk for complications during pregnancy [4]. In Yemen, previous reports indicate that hepatitis B is hyperendemic and infection with HBV and HCV is an important cause of chronic liver disease [5]. Investigating seroprevalence of hepatitis B surface antigen (HBsAg) and anti-HCV antibody in pregnancy in different settings is needed to prevent vertical transmission. Seroprevalence of these infections among pregnant women may be a good indicator of general population prevalence and a determinant of vaccination policy [6,7]. Pregnancy may be the only clinical access point for the antenatal population in limited resource settings such as Yemen. Successful interventions to prevent vertical transmission linked to prenatal and intrapartum testing have been demonstrated in a variety of limited resource settings [8,9]. Although, screening for HBsAg in pregnancy is recommended by the Royal College of Obstetricians and Gynaecologists (United Kingdom), American Congress of Obstetricians and Gynecologists, as well as many colleges of obstetrics and gynecology worldwide [10,11], screening of antenatal women for HBV is not a routine practice in Yemen. Also, routine vaccination of newborns is not widely available in Yemen.

Although much recent data are available on the epidemiology of HBV and HCV among pregnant women in different countries [12-16], few published data are available from Yemen [5]. Such data are fundamental for health planners and care givers for evidence-based intervention. The current study was conducted at the Al-Thawra hospital in Sana’a, Yemen to investigate the seroprevalence and associated risk factors for markers of HBV (HBsAg) and anti-HCV antibodies among pregnant women.

## Methods

### Hospital and patients

This work was conducted as part of Collaboration between Alzaiem Alazhari University, Khartoum, Sudan and Al-Thawra Hospital, Sana’a, Yemen in investigating communicable diseases during pregnancy [17]. A cross-sectional study was conducted at the Antenatal Care Clinic at Al-Thawra Hospital in Sana’a during November–December 2011. Al-Thawra Hospital is a tertiary care facility for local women who receive antenatal care at the hospital as well as for referrals from the other clinics and hospitals. All women with risk factors or obstetric/medical complications are referred to the hospital. However, the referral criteria are not strictly adhered to and many women without any significant complications are allowed to deliver at the hospital. After giving signed informed consent, women with a singleton baby were approached to participate in the study. Those with antepartum hemorrhage, hypertension and diabetes mellitus were excluded. A fixed questionnaire was applied to gather relevant sociodemographic characteristics (age, education and residence) and obstetric characteristics (gestational age and parity). Then the possible risk factors (e.g. history of surgery or blood transfusion, tattooing, and circumcision) for HBV were investigated. After centrifugation, serum samples were tested for HBsAg and anti-HCV using an ELISA (4^th^ generation).

### Statistics

A sample size of 400 patients was calculated based on a two-sided hypothesis test using Epiinfo with 80% power and 95% CI. Statistical analysis was performed using SPSS for Windows version 16.0 (SPSS Inc., Chicago, IL, USA) and all data were double-checked before analysis. The mean and proportion of the sociodemographic and clinical characteristics were calculated for the HBsAg and anti-HCV seropositive groups. Univariate and multivariate analyses were performed using seropositivity for HBsAg and anti-HCV as dependent variables and sociodemographic and clinical variables (age, residence, parity, circumcision, education, history of surgery, miscarriage, blood transfusion dental manipulation and traditional scar), as independent variables. A *p* value < 0.05 was considered significant.

### Ethics

Ethical approval was obtained from the Ethics Review Committee at Alzaiem Alazhari University, Sudan and at Al-Thawra Hospital, Sana’a, Yemen.

## Results

### General characteristics

Four hundred pregnant women were enrolled with a mean (SD) age of 26.8 (5.7) years. Seventy-nine and 77 of these pregnant women were primigravidae and secundigravidae, respectively. Around half (49.2%) of the women had educational level below secondary school (<9 years) and 197 (49.2%) were rural residents. None of the women were immunized against HBV. The sociodemographic details and clinical characteristics are shown in Table 1.

**Table 1:** Sociodemographic characteristics of pregnant women in Sana’a, Yemen seropositive for HBsAg and anti-HCV*

	**Total women (N = 400)**	**Women with HBSAg (N = 43)**	**Women with anti HCV(34)**
Age, years	26.8(5.7)	28.4(6.8)	27.2(6.0)
Gravidity	3.7(2.7)	4.6(3.1)	4.6(3.0)
Gestational age, weeks	27.1(6.9)	27.4(8.2)	26.9(5.8)
Education < secondary school	197(49.2)	26(60.5)	12(35.3)
Rural residence	112(28)	18(42.0)	12(35.3)
Blood transfusion	84(21.0)	10(23.3)	14(41.2)
Surgical operation	107(26.8)	13(30.2)	16(47.1)
Circumcision	22(5.5)	5(11.6)	1(2.9)
Home delivery	73(18.2)	13(30.2)	7(20.6)
Traditional scar	82 (20.5)	11(25.6)	12(35.3)
Dental manipulation	307(76.8)	7(16.3)	28(82.4)
History of miscarriage	152(38.0)	19(44.2)	17(50.0)

*Data are shown as mean (SD) or n (%) as applicable.

### HBsAg and anti-HCV seroprevalence

Among the 400 pregnant women enrolled in the study, HBsAg and anti-HCV was detected in 43 (10.8%; 95% CI: 8.0–14.0%) and 34 (8.5%, 95% CI: 6.0–11.5%), respectively. None of the patients were infected by both HBV and HCV.

### Risk factors for HBsAg or anti-HCV

Although multivariate analysis showed that circumcision was significantly associated with HBsAg seropositivity (OR = 3.3, 95% CI: 1.1–10.2; *p =* 0.03), age, gravidity and rural residency were significantly associated with HBsAg seropositivity in univariate analysis only. No other sociodemographic or clinical characteristics (history of home delivery, miscarriage, dental manipulation, surgery, traditional scar and blood transfusion) were significantly associated with HBsAg (Table 2).

**Table 2:** Univariate and multivariate analyses for possible risk factors for HBsAg among pregnant women in Yemen

**The variables**	**Univariate analysis**			**Multivariate analysis**		
	**O R**	**95**% **CI**	**P**	**O R**	**95**% **CI**	**P**
Age	0.9	0.8–0.9	0.04	0.9	0.9–1.0	0.9
Gravidity	0.8	0.8–0.9	0.03	0.9	0.7–1.1	0.6
Education < secondary school	0.6	0.3–1.1	0.1	1.0	0.6–1.5	0.9
Rural residence	2.0	1.1–3.5	0.03	1.9	0.9–3.9	0.06
Blood transfusion	1.1	0.6–2.6	0.6	1.0	0.6–1.5	0.9
Surgical operation	1.2	0.6–2.3	0.4	1.1	0.6–1.9	0.5
Circumcision	2.6	0.9–7.5	0.07	3.3	1.1–10.2	0.03
Home delivery	2.1	1.1–1.9	4.3	1.6	0.6–4.1	0.2
Traditional scar	1.3	0.6–2.8	0.3	1.0	0.6–1.7	0.7
Dental manipulation	1.6	0.7–3.8	0.2	1.4	0.5–3.4	0.4
History of miscarriage	1.3	0.7–2.5	0.3	0.9	0.4–2.1	0.8

In multivariate analysis, low parity (primigravidae and secundigravidae) (OR = 2.9, 95% CI: 1.1–8.4; *p =* 0.03) and education below secondary level (OR = 2.9, 95% CI: 1.2–7.0; *p =* 0.031) were significantly associated with anti-HCV seropositivity (OR = 3.3, 95% CI: 1.1–10.2; *p =* 0.03). History of blood transfusion, surgical operation, and traditional scarring were significantly associated with anti- HCV seropositivity in univariate analysis only. Other sociodemographic or clinical characteristics (age, residence, history of home delivery, miscarriage, circumcision, dental manipulation) were not significantly associated with anti-HCV seropositivity (Table 3).

**Table 3:** Univariate and multivariate analyses for possible risk factors for anti-HCV among pregnant women in Yemen

**The variables**	**Univariate analysis**			**Multivariate analysis**		
	**O R**	**95**% **CI**	**P**	**O R**	**95**% **CI**	**P**
Age	0.9	0.9–1.0	0.6	1.0	0.9–1.1	0.5
Primigravidae and secundigravidae	2.6	1.1–6.2	0.02	2.9	1.1–8.4	0.03
Education < secondary school	1.8	0.9–3.8	0.09	2.9	1.2–7.0	0.01
Rural residence	1.4	0.6–3.0	0.3	1.6	0.7–3.8	0.2
Blood transfusion	2.9	1.4–6.1	0.01	2.0	0.8–4.8	0.1
Surgical operation	2.7	1.3–5.5	0.01	1.6	0.6–4.0	0.2
Circumcision	0.4	0.6–3.8	0.5	0.4	0.5–3.9	0.4
Home delivery	1.1	0.5–2.8	0.6	1.1	0.4–3.1	0.7
Traditional scar	2.2	1.1–4.8	0.03	1.0	0.6–1.4	0.9
Dental manipulation	1.4	0.5–3.6	0.4	1.2	0.4–3.4	0.6
History of miscarriage	1.7	0.8–3.4	0.1	1.2	0.5–2.8	0.5

## Discussion

These are believed to be the first published data on HBsAg and anti-HCV among pregnant women in Yemen. The main finding of the study was the high prevalence of HBsAg and anti-HCV (10.8% and 8.5%) among pregnant women in this setting. The prevalence of HBsAg and anti-HCV among these pregnant women was higher than the prevalence of these markers among healthcare workers in large hospitals in Sana’a [54/543 (9.9%) and 19/546 (3.5%)] [18], Ibb (1.81% and 1.99%) [19], and Aden [20].

Recent reports have documented a lower prevalence of HBsAg and anti-HCV among pregnant women in different countries; for example, 4.0% and 6.4% in Egypt [12], 4.6% and 7.0% in Pakistan [13], 2.8% and 0.1% in Turkey [14], 1.53% and 0.31% in Afghanistan [15], and 5.6% and 0.3% in Sudan [16]. A finding similar to that by Elsheikh *et al.*, in this study is that none of the patients were infected by both HBV and HCV [16].

The current study showed that circumcision was significantly associated with HBsAg seropositivity, and low parity and lower education levels were significantly associated with anti-HCV seropositivity. Female circumcision, which is usually performed at home by midwives or health workers at young ages, might be the source of infection in these women. Recently, it has been observed that previous history of surgery, multiple injection therapy, and blood transfusion was a risk factor among anti-HCV and HBsAg-positive pregnant women [14], and high parity, older age and blood transfusion were independently associated with HCV among pregnant women [21,22]. However, none of the known risk factors was found to be significantly associated with the HCV infection among pregnant Indian women [23]. Other risk factors such as early age of sexual debut, history of multiple sexual partners, and past history of sexually transmitted infection were found to be significantly associated with HBV among pregnant women [24]. As a result of social and religious reasons, these factors are difficult to be investigated in Yemen. Perhaps if these risk factors were investigated, different results might have been obtained.

In spite of high prevalence of HBsAg and HCV in the current study, some points need to be mentioned. The population investigated consisted only of women able to access antenatal care, and the prevalence reported here may have underestimated the true prevalence among pregnant women in the community. Furthermore, these women presented to a tertiary hospital, therefore, they cannot represent Yemen as a whole. The prevalence is high when compared to the general population and this might be explained by the population being over-represented. As mentioned above, there may have been under-reporting of risky behaviors or other socially undesirable responses in this survey. The majority of the mothers were from low socioeconomic backgrounds; therefore, it is difficult to suggest that the infection was more prevalent in those with a lower educational level. These women were not screened for hepatitis D virus, HIV and other sexually transmitted diseases, and their partners were not screened for HBV and HCV. Unfortunately it was difficult to follow-up these women till delivery to establish vertical transmission and its associated factors.

## Conclusions

The results of this study suggest that HBsAg and anti-HCV have high prevalence among pregnant women. Circumcision was a risk factor for HBsAg, and low parity and lower education levels were risk factors for anti-HCV.

## Competing interests

The authors declare that they have no competing interests.

## Authors’ contributions

EAM, SMB and IA were involved in study conception and design. DAR and GIG were involved in acquisition, analysis and interpretation of data. All the authors were involved in drafting the manuscript and its critical revision. All authors read and approved the final manuscript.

## Pre-publication history

The pre-publication history for this paper can be accessed here:

http://www.biomedcentral.com/1471-2393/13/127/prepub
